# The role of mood disorders in the longitudinal course of obsessive-compulsive disorder: Preliminary data from a 20-year prospective follow-up study

**DOI:** 10.1192/j.eurpsy.2021.374

**Published:** 2021-08-13

**Authors:** S. Bramante, A. Borsotti, S. Rigardetto, G. Maina

**Affiliations:** 1 Neuroscience, università degli studi di torino, torino, Italy; 2 Scdu Psichiatria, Azienda Ospedaliero Universitaria San Luigi Gonzaga, Orbassano, Italy

**Keywords:** obsessive compulsive disorder, follow-up, mood disorders, remission

## Abstract

**Introduction:**

Although OCD is believed to have a chronic course, little research has been conducted on this, and there are discrepant findings. Studies over the last years have found that a significant proportion of patients with OCD shows symptomatic remission over long term, however there are significant variations in sampling, clinical characteristics, follow-up, and outcome assessments.

**Objectives:**

The present prospective study aims to examine rates of OCD remission after 20 years of follow up and to explore demographic and clinical predictors of remission.

**Methods:**

The study sample consists of adult patients with a principal OCD diagnosis and Y-BOCS total score ≥16, who have been referred to the Department of Neuroscience, University of Turin (Italy). OCD symptoms were assessed every 5 years over the 20-year follow-up period. Course data were examined using standard survival analysis methods; Cox proportional hazards regression was used to estimate relative hazards for predictors of remission.

**Results:**

There were 360 participants in the study. At year 20, the 28.7 % of the total sample showed OCD remission. Predictor of remission were female gender, lower Y-BOCS mean scores at study entry, longer duration of illness and comorbidity with major depressive disorder. No specific predictors of full remission were found. Lower Y-BOCS mean scores and comorbid bipolar disorder predicted partial remission.

**Conclusions:**

This study suggests that a significant proportion of patients with OCD shows remission. Future studies are needed to clearly identify predictors of remission.

**Disclosure:**

No significant relationships.

